# Transcription Regulation of Sex-Biased Genes during Ontogeny in the Malaria Vector *Anopheles gambiae*

**DOI:** 10.1371/journal.pone.0021572

**Published:** 2011-06-30

**Authors:** Kalle Magnusson, Antonio M. Mendes, Nikolai Windbichler, Philippos-Aris Papathanos, Tony Nolan, Tania Dottorini, Ermanno Rizzi, George K. Christophides, Andrea Crisanti

**Affiliations:** 1 Department of Life Sciences, Imperial College London, London, United Kingdom; 2 Department of Experimental Medicine, University of Perugia, Perugia, Italy; 3 Istituto di Tecnologie Biomediche, Consiglio Nazionale delle Ricerche, Milano, Italy; Institute of Genetics and Molecular and Cellular Biology, France

## Abstract

In *Anopheles gambiae*, sex-regulated genes are responsible for controlling gender dimorphism and are therefore crucial in determining the ability of female mosquitoes to transmit human malaria. The identification and functional characterization of these genes will shed light on the sexual development and maturation of mosquitoes and provide useful targets for genetic control measures aimed at reducing mosquito fertility and/or distorting the sex ratio.

We conducted a genome wide transcriptional analysis of sex-regulated genes from early developmental stages through adulthood combined with functional screening of novel gonadal genes. Our results demonstrate that the male-biased genes undergo a major transcription turnover starting from larval stages to adulthood. The male biased genes at the adult stage include a significant high number of unique sequences compared to the rest of the genome. This is in contrast to female-biased genes that are much more conserved and are mainly activated during late developmental stages.

The high frequency of unique sequences would indicate that male-biased genes evolve more rapidly than the rest of the genome. This finding is particularly intriguing because *A. gambiae* is a strictly female monogamous species suggesting that driving forces in addition to sperm competition must account for the rapid evolution of male-biased genes. We have also identified and functionally characterized a number of previously unknown *A. gambiae* testis- and ovary-specific genes. Two of these genes, zero population growth and a suppressor of defective silencing 3 domain of the histone deacetylase co-repressor complex, were shown to play a key role in gonad development.

## Introduction

Mosquitoes (Diptera:Culicidae) are a heterogeneous group of insect species that show substantial genetic and biological differences. Such differences reflect on their ability to colonize distinct environmental niches and their capacity to transmit diseases to animals and humans. Some mosquito species of the genus *Anopheles* are responsible for the transmission of human malaria in tropical and subtropical regions of the world, whereas *Aedes* mosquitoes transmit worldwide a number of pathogenic viruses including yellow fever, several kinds of encephalitis and dengue fever. Notably only female mosquitoes are able to function as disease vectors as they must feed on blood to produce their progenies. Females differ from males in several morphological, biological and behavioural traits that are critical for determining their ability to transmit a disease. These include feeding preference, feeding behaviour, longevity and susceptibility to parasite and/or virus infections. *A. gambiae* mosquitoes in particular combine an exclusive anthropophagic feeding preference with a high susceptibility to *Plasmodium falciparum* infection, which make them a very efficient vector species of human malaria [1], [2].

In *A. gambiae,* the male is the heterogametic sex, and sequence analysis and experimental data indicate that the Y chromosome of *A. gambiae* is, to a large degree, composed only of repetitive sequences and transposon relics with very few, if any, transcriptionally active genes [3]. Therefore, despite important differences in the morphology, the physiology and the behaviour of male versus female *A. gambiae* mosquitoes, the gene repertoires of the two sexes must be nearly identical. This implies that most of the phenotypic traits contributing to *A. gambiae* sexual dimorphism are determined, similarly to what has been observed in several other species, by the differential expression (sex-bias) of genes that are present in both sexes [4], [5], [6]. An analysis of the sex-biased genes in *A. gambiae* is therefore critical to reveal the molecular basis for the vectorial capacity of this mosquito. Available information on sex-biased genes does not take into account the transcription at early developmental stages such as embryos, larvae and pupae when sex differentiation is committed and the process of sexual maturation initiates [7], [8].Knowledge on the nature of the primary signals that regulate sexual dimorphism established during these early stages is of fundamental biological importance and can facilitate the development of genetic vector control measures targeting the mosquito population reproduction capability. In addition, comparative analysis of sex-biased genes in several insect species has revealed unique features that are relevant for understanding the process of evolution and speciation [9], [10]. In adult male *Drosophila*, sex-biased genes associated with sexual traits and reproduction show an unusually rapid sequence evolution compared to the rest of the genome, as evidenced by the high ratio of non synonymous to synonymous mutations and the low frequency of one-to-one orthologues in the genome of related species [9]. *A. gambiae* females are monogamous [11], and therefore this species represents a valuable model to investigate the evolution of sex-biased genes in the absence of sperm competition.

We carried out a genome-wide transcriptional, phylogenomic and functional analysis on sexregulated genes from early developmental stages, including one-day old larvae and pupae, as well as adult male and female mosquitoes. We have utilized a novel transgenic *A. gambiae* line that, for the first time, allows the *in-vivo* identification of male and female *A. gambiae* mosquito larvae immediately after hatching on the basis of a strong male-biased eGFP-expression.

## Results

### Ontogeny of sex-biased transcription in *A. gambiae*

A transgenic *A. gambiae* line *dsx*–eGFP was generated that expresses high levels of eGFP selectively in male larvae from early developmental stages throughout adulthood thereby offering the possibility to reliably determine the sex of mosquitoes from early developmental stages throughout adulthood (Figure S1). We confirmed integration into a single genomic location and the absence of physical or fitness related phenotypes in this line (data not shown).

**Figure 1:**
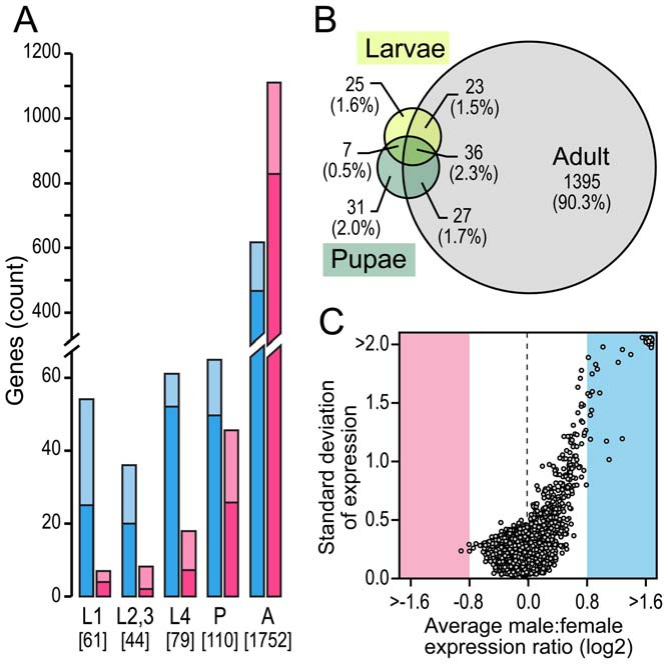
Developmentally regulated *A. gambiae* sex-biased genes. (A) Number of female- (pink) and male- (blue) biased genes transcribed at the larval, pupal and adult stages. The bars are split in light and dark colours to arrange sex-biased genes according to two different thresholds of male:female log_2_ expression ratios +/− 0.8 and +/− 1.6 respectively. (B) Venn diagram showing the relationship between sex-biased transcription and development. Larval stages were pooled together to simplify their visualization. The relative percentage of sex-biased genes respect to a total of 1544 genes with transcription values at all stages examined is shown in parenthesis. (C) Variance of gene expression across different developmental stages at increasing male:female expression ratios. The average male:female expression ratio of each gene at all stage examined is plotted against the standard deviation of its expression. The male-biased expression is the most relevant turnover of sex-linked expression across mosquito development though at the adult stage female-biased genes are more numerous than the male-biased ones. See also Figure S1 and Tables S1, S2, S3.

Male and female RNA extracted from *dsx*–eGFP sexed larvae (1^st^ instar, late 2^nd^/early 3^rd^ instar and 4^th^ instar), pupae and virgin non-blood-fed 3 day old adult mosquitoes was competitively hybridized onto the MMC-2 microarray, a PCR amplicon microarray platform that encompasses 7246 genes of the current *A. gambiae* gene build. A total of 6142 of these genes showed reproducible transcription values (Table S1). Genes were considered to be either male or female-biased when the value of the log_2_ male/female expression ratio was higher than 0.8 (1.73-fold) and lower than -0.8 (0.57-fold), respectively. According to these criteria we found 1884 genes (30.7%) to have a male- or female- transcription bias in one or more of the developmental stages examined (Table S2). We observed major changes in the relative proportion of female- and male-biased genes and their differential expression ratio values across the various developmental stages (Figure 1A). The number of sex-biased genes increased from as few as 61 (1% of the total number of genes examined) at the stage of 1^st^ instar larvae to 1752 (28.5%) in adult mosquitoes, 1309 (74.7%) of which presented more than 2-fold difference between sexes (Figure 1A). At the adult stage, female-biased genes were much more abundant (1123 genes) than male-biased genes (629 genes) whereas at the larval and pupae stages their ratio was reversed. At all larval stages examined, more than 80% of the sex-biased genes were male-biased and at least 39% had a male/female transcription ratio value higher than 2-fold (Figure 1A). Noticeably, the proportion of male-biased genes to the total of sex-biased genes increased with the value of male/female transcription ratios reaching nearly 100% at values higher than 3-fold. While at the larval stages we detected a total of 91 sex-biased genes, only 36 maintained transcription bias across the larvae, pupae and adult stages (Figure 1B and Table S3). A substantial fraction of these genes (92%) are male-biased and include genes with orthologues involved in spermatogenesis. In general male-biased genes showed a more variable expression profile, indicating a greater contribution to the overall expression divergence between males and females across mosquito development (Figure 1C).

We assessed the quality of these data by comparing the fluorescence hybridization signal of both male and female 1^st^ instar larvae RNA onto the MMC-2 microarray with the number of transcript traces generated by 454 sequencing of the same mRNA (Figure S2). This analysis showed a good correlation between the microarray and the 454 transcriptome sequencing datasets (Spearman correlation: 0.448, P value<0.001; Spearman correlation: 0.455, P value<0.001). We further validated the microarray data by examining in qRT–PCR experiments the expression profiles of three genes (AGAP003087, AGAP006241 and AGAP001416) with distinct sex-biased profiles across mosquito life stages (Figure S3). In addition, we compared our expression data collected at the adult stages to available data from studies performed with different microarray platforms [8] and found a high degree of correlation between studies (Spearman correlation: 0.341, P value<0.001; Spearman correlation: 0.481, P value<0.001) (Figure S4).

### Transcriptional profile analysis of sex-biased genes

**Figure 2:**
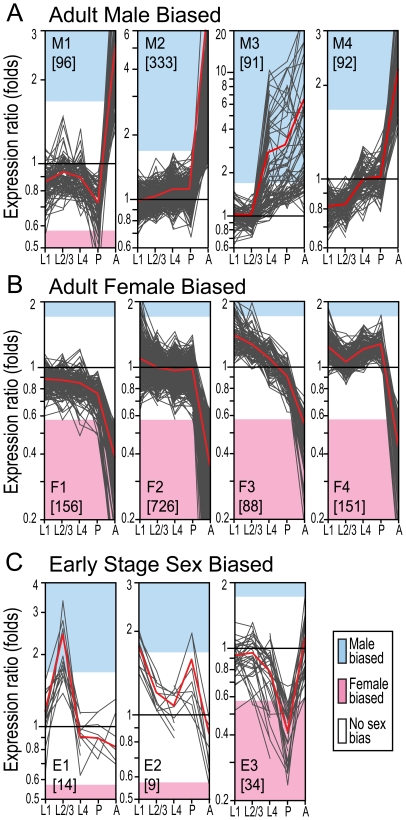
Sex-biased developmental transcription programmes of co-expressed genes. The sex-biased genes with transcription values for at least three of the five developmental stages analysed (1^st^ instar larvae (L1), 2^nd/^3^rd^ instar larvae (L2-3), 4^th^ instar larvae (L4), pupae (P), adults (A)) are arranged in co expression K-means clusters. The number of genes included in each cluster is indicated in brackets. The clusters are arranged in three groups according to the average sex-bias profile at the adult stage. Group A (M1-M4) and B (F1-F4) contain genes that have distinct developmental transcription profiles but have a male- and female-bias respectively at the adult stage. Group C (E1-E3) contains genes that are sex-biased (either male or female) at early developmental stages but not sex-biased in adult mosquitoes. See also Figure S6 and Tables S4, S7.

We isolated temporally co-regulated sex-biased genes using a combination of quality threshold and K-means cluster analysis. This analysis yielded 11 gene clusters that differed from each other on the basis of their temporal and sex-biased transcription profile (Table S4). The clusters M1 to M4 contained genes showing a similar male-bias transcription profile in the adult stage, but differing at the earlier developmental stages (Figure 2A). Clusters F1 to F4 contained adult female-biased genes, whereas clusters E1 to E3 encompassed genes that were either male- or female-biased at some of the early developmental stages examined but did not exhibit sex-biased transcription in the adult stage (Figure 2B and C).

**Figure 3:**
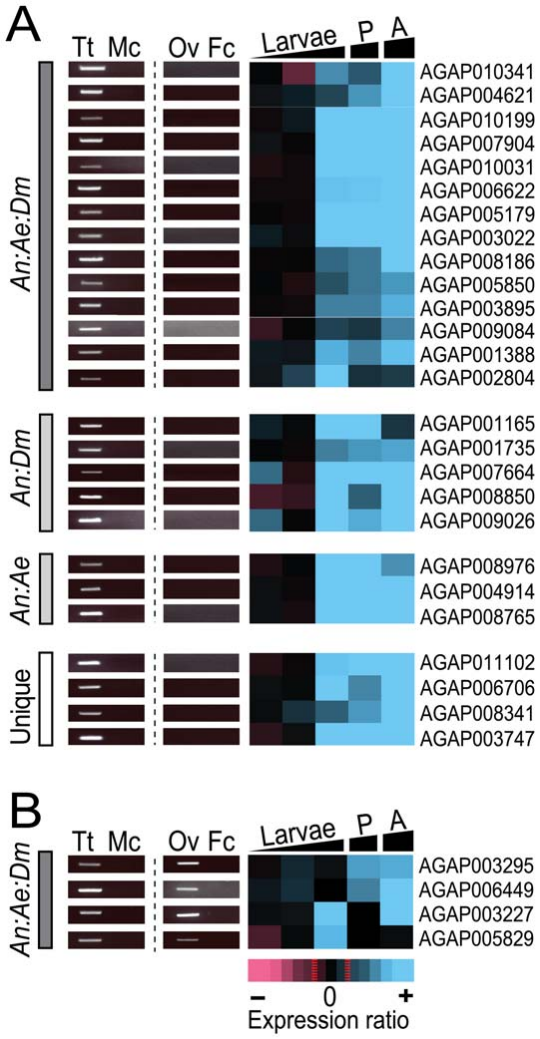
Tissue transcription analysis of M3 cluster components. (A) The genes of cluster M3 transcribed exclusively in the testis and (B) in both testis and ovaries are arranged on the basis of their transcription profile during ontogeny (1^st^ instar larvae, 2^nd/^3^rd^ instar larvae, 4^th^ instar larvae, pupae (P) and adults (A)) and analyzed for the presence of identifiable *A. gambiae* (*An*) orthologues in the genomes of *D. melanogaster* (*Dm*) and *Ae. aegypti* (*Ae*). The male:female transcription ratio values are translated into a colour code eisengram ranging from blue (top at 3.0 log_2_) to pink (bottom at -3.0 log_2_) showing different levels of male- and female-biased transcription intensity respectively. For each gene RT-PCR experiments were performed on the testes (Tt), carcass of adult males (Mc), ovaries (Ov) and carcass of adult females (Fc). See also Tables S5, S6, S8.

Genes in cluster M3 exhibited a highly conserved sex-biased transcription pattern from the 4^th^ instar larval stage to adulthood and generally had very high expression ratio values, in several cases reaching up to 20-fold differential expression between the two sexes. Detailed analysis showed that cluster M3 was enriched in genes with orthologues in several other species including *D. melanogaster* and *Mus musculus* with known functions in testis and spermatogenesis. AGAP004754 and AGAP005850 have *D. melanogaster* orthologues, which are involved in sperm individualization (*Nd*) [12] and sperm motility (*GAS8*) [13] respectively. AGAP008275, AGAP008765, AGAP010199 and AGAP010786 have mamalian orthologues that are involved in sperm motility (*Tekt2*)) [14], male fertility (*Tssk6*) [15], male meiosis (*Rsph1*) [16] and spermatogenesis (*Spag1*) () [17] respectively. Prompted by these distinct features of cluster M3, we searched for additional novel testis- transcribed genes by performing an RT-PCR analysis on testis, ovaries and carcasses for each of the 91 genes found in this cluster. This analysis revealed the presence of 26 novel genes transcribed exclusively in the testis (Figure 3 and Table S5). Of those, only four (AGAP011102; AGAP006706; AGAP008341; AGAP003747) were unique to *A. gambiae* and did not have identifiable orthologues in other species. Four other genes of cluster M3, though being male-biased, were transcribed in both the testis and ovaries but not in the carcass (Figure 3 and Table S5). These genes were AGAP006449, showing sequence homology to a component of the histone deacetylase co-repressor complex (Table S5), AGAP003295, AGAP005829 and AGAP003227 (Figure 3).

As anticipated clusters F1-F4 contained several known female-specific genes transcribed in the gut and salivary glands. These clusters showed a temporal transcription pattern that was different from that of M3, indicating that male and female gametogenesis processes encompass distinct transcription programmes (Figure 2). Consistent with this finding, two known *Anopheles* ovary-specific genes, AGAP003545 (*oskar*) and AGAP006098 (*nanos*), showed female-biased transcription only at the adult stage [18], [19]. They were found in cluster F2 and F3 respectively. To search for additional ovary-specific genes, we investigated, by RT-PCR, the transcription profile of 76 genes chosen amongst those showing the highest female-bias values at the adult stage (Table S6). We found two previously uncharacterized *A. gambiae* ovary-specific genes: AGAP010219 and AGAP003087. The former is an orthologue of the *D. melanogaster mcm-6* gene, which is involved in DNA replication and chorion gene amplification [20], and the latter that lacked known functional domains had identifiable orthologues in many insect genomes and exhibited the strongest adult female-bias among the ovary-specific genes. Cluster E3 showed a female-biased transcription only at the pupal stage. At earlier developmental stages and later in adult mosquitoes these genes did not show any sex transcription bias. Eleven out of the 34 genes (32.3%) of cluster E3 encode proteins known to play a role in the insect immune system (DEF1, CEC2, CEC3, CASPS3, CLIPB17, ML3, TEP3, TEP4, TEP5, TEP9, TEP10 and TEP12) [21]. Furthermore, the genes AGAP000275, AGAP001345, AGAP001657, AGAP002227 and AGAP006546 encode reduction-oxidation reaction, apoptosis or detoxification domains that are also frequently found in proteins known to play a role in the insect immune system [1].

### Compositional analysis of transcriptional programs

The functional significance of the distinct transcriptional programs was assessed through the analysis of gene ontology (GO) terms frequencies as well as the occurrence of known protein domains as classified by InterPro signatures (Table S7 and table S8). Cluster M3 showed a significant overrepresentation (*P*  =  <0.001) of the cellular component related GO term “microtubule” in agreement with the observation that this cluster contained a great number of genes involved in spermatogenesis. Microtubules, among other roles, play an important role during mitosis and meiosis, and function as the building blocks of the sperm axoneme that is essential for spermatozoa motility [22]. InterPro composition analysis also revealed that cluster M3 contained all three *A. gambiae* tektin genes (Table S8) that are known to play a role in the function and assembly of the sperm cell flagella [23]. The female-biased clusters F1 and F2 were significantly enriched for GO terms related to mRNA translation including “ribosome, translation ribonucleoprotein and ribosome constituent” (*P*  =  <0.001) (Table S7). This temporal expression pattern coincides with the development and differentiation of female-specific tissues as shown by the dramatic over-representation of female-biased genes in these stages. It is therefore not surprising that these clusters show a significant overrepresentation of terms associated with the process of translation. InterPro domain composition analysis revealed a significant high frequency of members of the Brix protein family (*P*  =  <0.001) and protein synthesis factors (*P*  =  <0.001) respectively consistent with the GO term analysis in cluster F1 and F2 (Table S7 and table S8). Cluster E3 mainly contained GO terms associated with the immune response function such as “defence response”, “innate immune response” and “immune response”. Accordingly Interpro domain distribution showed a high frequency of proteins with domains involved in immunity such as “alpha-2-macroglobulin” and “cecropin” [24].

### Sequence evolution of *A. gambiae* sex-biased genes

**Figure 4:**
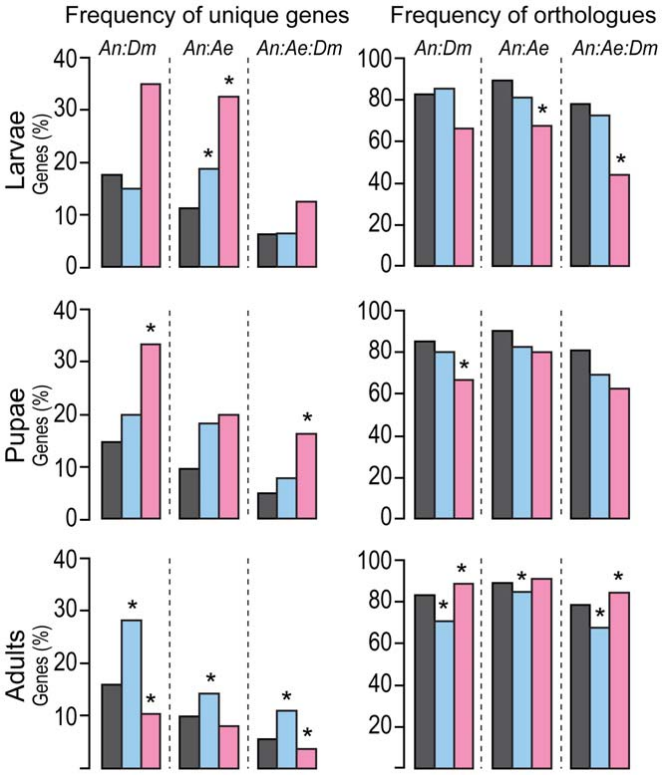
Phylogenomic analysis of *A. gambiae* sex-biased genes. Percentages of male- (blue) and female-biased (pink) genes identified at the stage of larvae, pupae and adults that are either unique (left panels) or have identifiable orthologues (right panels) when comparing the genomes of *A. gambiae* with *D. melanogaster* (*An:Dm*), *A. gambiae* with *Ae. Aegypti* (*An:Ae*) and *A. gambiae* with *Ae agypti* and *D. melanogaster* (*An:Ae:Dm*). Differences in the percentage of either unique sequences or orthologues compared to those observed in the subset of all genes transcribed at a particular stage (grey) were statistically evaluated by Bonferroni corrected hypergeometric distribution (P<0.05, one asterisk). See also Tables S9, S10.

Due to the lack of suitable *Anopheles* genome sequences to assess non-synonymous and synonymous substitution (dN/dS) ratios we utilized two alternative and complementary approaches to calculate the rate of evolution of sex-biased and non-sex-biased *Anopheles* genes. First, we assessed across development the frequency of *A. gambiae* male- and female-biased genes that are either species-specific (unique) or have identifiable orthologues in the genomes of *Ae. aegypti* and/or *D. melanogaster*. We then compared the frequency of synonymous codons in both the total and the sex-biased genes on the assumption that non-random distributions would reflect differences in the evolution of male- and female-biased genes [25]. Indeed, even if the positive selection for synonymous codon usage is expected to be relatively weak, it has been shown in *D. melanogaster* that the utilization of optimal codons can affect the fitness of insects at the phenotypical level, with the possessors of biased genes enjoying some fitness advantages [26]. Our data revealed that the frequencies of unique sex-biased and non-biased genes were different depending on the developmental stage examined (Figure 4 and Table S9). At early developmental stages, the female-biased genes showed a higher frequency of unique sequences compared to the overall set of genes analysed, which was not observed for male-biased genes. On the contrary, in adult mosquitoes the percentage of male-biased genes without identifiable orthologues in the genomes of *Ae. aegypti* and *D. melanogaster* was significantly higher than the rest of the genome (*P*  =  <0.001) (Figure 4 and Table S9). The distribution bias of synonymous codons was measured using two metrics, the effective number of codons (ENC) and the frequency of optimal codons (Fop). The analysis of the distribution of synonymous codons revealed that the codon usage of adult male-biased genes was significantly more random (ENC *P*  =  <0.001, Fop *P*  =  <0.001) (or less biased) than that of adult female- and non-sex-biased genes (Table 1). A corresponding decrease in codon bias was observed for female-biased genes at the larval stages (ENC *P* = 0.006, Fop *P* = 0.003) (Table 1). Furthermore, we found a negative correlation between codon bias and the level of expression of male-biased genes (Figure S5) indicating that the observed differences in codon bias are not a consequence of an adaptive response of codon bias to match the optimal tRNA pools for gene expression, neither are they an artefact caused by the difference in gene length as adult male-biased genes are smaller than the female biased ones. These findings together strongly suggest that adult male-biased *A. gambiae* genes evolve more rapidly than the rest of the genome.

**Table 1:** Levels of codon bias for sex-biased genes across developmental stages.

		N total	Length	ENC	F_OP_
**Larvae**	Total	4963	1564	45.65	0.67
	Male biased	113	2406 (P<0.001)	46.12 (P = 0.341)	0.65 (P = 0.035)
	Female biased	27	1283 (P = 0.047)	49.56 (P = 0.006)	0.60 (P = 0.003)
**Pupae**	Total	3674	1534	44.85	0.69
	Male biased	41	1558 (P = 0.596)	47.09 (P = 0.059)	0.62 (P = 0.072)
	Female biased	36	941 (P<0.001)	43.72 (P = 0.461)	0.69 (P = 0.461)
**Adult**	Total	4121	1534	45.23	0.68
	Male biased	503	965 (P<0.001)	49.85 (P<0.001)	0.59 (P<0.001)
	Female biased	954	1547 (P<0.001)	43.36 (P<0.001)	0.71 (P<0.001)

N indicates number of genes; Length indicates the mean number of base pair nucleotides for each category; ENC indicates the mean effective number of codons for each category; Fop indicates the mean frequency of optimal codons for each category. P-value for Kolmogorov-Smirnov two-sample tests comparing values for male or female biased genes to the total number of genes in each category are presented within brackets. See also Figure S5.

Notably, our analysis reveals that not all male-biased genes appear to evolve at the same rate. Phylogenomic comparison within individual clusters (Figure S6 and Table S10) showed that the transcription profile of male-biased genes correlated with the frequency of unique sequences. The male clusters M1 and M2 contained a significantly higher percentage of unique sequences when compared with the genomes of *Ae. aegypti* (M1 *P* = 0.03, M2 *P*  =  <0.001)and *D. melanogaster* (M1 *P*  =  <0.001, M2 *P*  =  <0.001). In contrast, genes of cluster M3 (enriched for genes involved in spermatogenesis) were more conserved in terms of orthology (*Ae. aegypti P* = 0.12, *D. melanogaster P* = 0.11) (Figure S6 and Table S10). The female and the early stage clusters did not show any significant bias in the proportion of unique gene sequences. Such difference in evolution rates between male-biased transcriptional programs may help explain why it has not been detected in a previous study [8].

**Figure 5:**
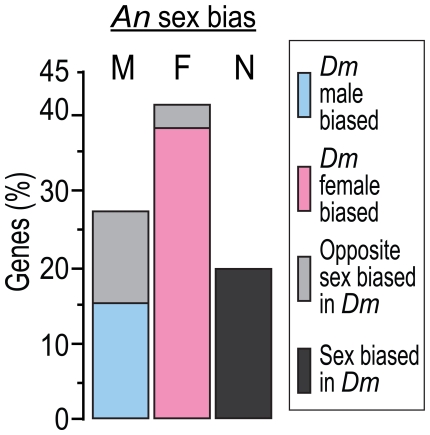
Transcription of sex-biased genes in *A. gambiae* and *D. melanogaster*. The transcription profile of a total of 118 male- (M), 334 female- (F) and 1343 non sex-biased (N) *A. gambiae* genes was compared to that of their one-to-one *D. melanogaster* orthologues. The bars show the proportion of *A. gambiae* sex-biased genes with *D. melanogaster* orthologues showing either a conserved male (blue), female (pink) sex-bias or a reversed (light gray) transcription pattern (i.e. male-biased genes becoming female-biased and vice versa). The proportion of *A. gambiae* non sex-biased genes with sex-biased *D. melanogaster* orthologues is also shown (dark grey). See also Table S11.

The comparison of *A. gambiae* and *D. melanogaster* microarray transcription revealed that only 18 (15%) out of 118 *A. gambiae* male-biased genes maintained the same sex-biased expression pattern of their corresponding fruit fly orthologues whereas a much higher proportion of female-biased genes, 126 out of 334 (38%), showed a conserved transcription pattern across the two species (Figure 5). Further analysis showed that the majority of the male-biased genes with a conserved transcription profile were selectively expressed in the *D. melanogaster* testis (15 out of 18 genes) (Table S11). We also observed that 273 *A. gambiae* non-sex-biased genes were reported to have a sex-biased transcriptional profile in *D. melanogaster*.

### Functional analysis of sex-biased *A. gambiae* genes

Experiments validating the efficiency of embryonic RNAi in Anopheles were performed by targeting the DsRed transgenic marker gene. Approximately 50% of the surviving DsRed-dsRNA-injected mosquitoes showed either a complete or partial absence of DsRed fluorescence, which lasted throughout the larval stages (Figure S7). These results indicate that injecting dsRNA into the embryo is a feasible way of knocking down developmentally important genes in *A. gambiae,* even if with limited penetrance.

**Figure 6:**
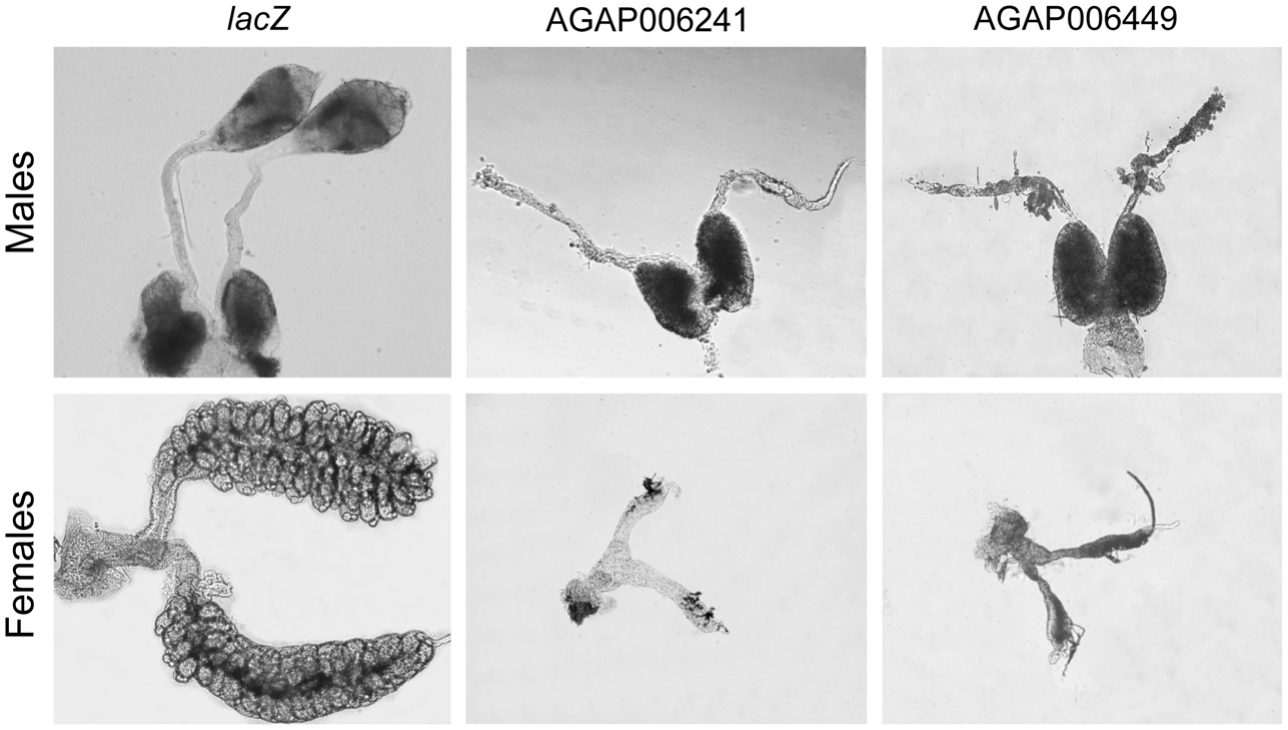
Development of internal reproductive organs in dsRNA injected mosquitoes. Micrographs of dissected male (upper panels) and female reproductive tracts (lower panels) of adult individuals that had developed from *lacZ* dsRNA, AGAP006241-dsRNA -and AGAPP006449 –dsRNA injected embryos.

We then performed RNA interference (RNAi) experiments in *A. gambiae* embryos for 8 male-biased genes with unknown function found in cluster M3, including 7 genes transcribed exclusively in the testis and 1 gene transcribed in the gonads of both sexes. These genes were selected because they encoded DNA binding proteins, transcription factors and molecules that could be involved in signalling pathways (Table 2). At least 800 embryos were injected with each of the double-stranded RNA (dsRNA) species. Replicate injection experiments were performed when a visible developmental phenotype was observed. Between 4% and 14% of the injected embryos developed into adults that were examined and dissected to analyse tissue and organ development. While the injection of dsRNA targeting the control bacterial gene *lacZ* did not cause any anomaly in the development of external and internal sexual organs, 6 females and 7 males out of 144 surviving mosquitoes injected with dsRNA targeting the gene AGAP006449 showed a severe developmental arrest of the gonads (Table 2). This gene encodes a yet uncharacterized protein containing a suppressor of defective silencing 3 (SDS3) domain of the histone deacetylase co-repressor complex. Morphological analysis showed that in the adult males the testis failed to develop and no signs of spermatogenesis were observed whereas the vas deferens and the accessory glands were not affected (Figure 6). The adult females showed in the place of the ovaries a pair of filamentous structures, the oviducts, of varying lengths that were completely devoid of follicles (Figure 6). Apart from the developmental arrest of the gonad, AGAP006449 dsRNA-treated mosquitoes appeared normal. Males and females developed clear dimorphic phenotypes and the external sexual genitalia and the internal organs, with the exception of the gonads, did not show any apparent anomaly. The lack of gonads indicated that the corresponding mosquito gene must play a critical role in the development of both testis and ovaries. Accordingly transcription analysis performed by RT-PCR on dissected organs and tissues showed that AGAP006449 was selectively transcribed in both the male and female gonads (Table S5). We conducted an additional set of RNAi experiments targeting AGAP006241 an innexin gene with homology to *D. melanogaster zero population growth* (*zpg*) (Table 2). In *D. melanogaster*, this gene encodes a germline-specific gap-junction protein (Innexin 4) required for the survival of early-differentiating germ cells during gametogenesis in both sexes [27]. Mosquitoes injected with dsRNA against AGAP006241 showed a phenotype very similar to that observed when silencing AGAP006449, characterized by a severe impairment of gonad development in both sexes (Figure 6) We performed qRT-PCR experiments with AGAP006449 and AGAP006241 dsRNA injected mosquitoes in order to determine knockdown efficiency following embryonic RNAi. We found that the transcript levels of both AGAP006449 and AGAP006241 were reduced as compared to *lacZ* controls (Figure S8). We believe that incomplete knockdown was the reason why a fraction of individuals injected with AGAP006241 and AGAP006449 dsRNA still developed gonads. Indeed an incomplete penetrance of the phenotype was also observed in the experiments targeting DsRed. This could be due to the distribution of dsRNA in areas of the embryo not coinciding with expression of the target gene or variability arising from the injection procedure of *A. gambiae* embryo injection which is known to be problematic.

**Table 2:** RNAi mediated targeting of selected sex-biased genes selectively transcribed either in the testis only or in both the testes and the ovaries.

				Gonad development anomalies	
Gene ID	Protein domain ID & Description	N. embryos injected	N. mosquitoes eclosed	Male	Female
**Testis –transcribed genes**					
AGAP001388	IPR001275: DM DNA-binding	1089	87	0/41	0/46
AGAP010341	IPR008271: Serine/threonine protein kinase, active site	878	73	0/39	0/34
AGAP001165	IPR007087: Zinc finger, C2H2-type	810	112	0/59	0/53
AGAP007664	IPR000956: Stathmin	840	34	0/18	0/16
AGAP008765	IPR008271: Serine/threonine protein kinase, active site	1637	111	1*/54	0/57
AGAP008976	IPR002373: cAMP/cGMP-dependent protein kinase	857	58	0/30	0/28
AGAP008341	IPR008271: Serine/threonine protein kinase, active site	876	48	0/23	0/25
**Testis and ovaries transcribed genes**					
AGAP006241	IPR000990: Innexin	1621	126	27/60	34/66
AGAP006449	Pfam 08598: SDS3 domain	1583	144	7/73	6/71
**Control**					
*LacZ*		1214	87	0/42	0/45

*A single individual showed one normal testis on one side and a duplicated testis on the other side.

## Discussion

Transcriptome analysis revealed that the development of male and female mosquitoes is accompanied by profound changes in the global patterns of sex-biased gene expression throughout life and between different developmental stages that include groups of functionally related genes such as genes mainly expressed in reproductive tissues (testis genes, cluster M3) and immunity-related genes (cluster E3). Phylogenomic comparison of co-regulated genes together with codon usage analysis revealed that groups of sex-biased genes are evolving at different rates. The adult male-biased genes show a significant lower frequency of one-to-one orthologues in the genomes of both *D. melanogaster* and *Ae. aegypti*, compared to non-biased and female-biased genes. This finding indicates that male-biased genes are much less conserved than the rest of the genome and support the notion that they evolve more rapidly under a sex-specific selection pressure. The notion that male-biased genes have significantly higher rates of evolution than the rest of the genome is also supported by the observation male-biased genes show a strong and consistent pattern of reduced codon bias. Previous microarray studies in *Drosophila*, *Caenorhabditis* and mammals have shown that male-biased genes, and especially genes expressed in sperm, evolve more rapidly compared to the rest of the genome [28]. The rapid evolution of genes expressed in sperm is likely to be the result of sperm competition, sexual selection and sexual conflict [28]. While the polygamous mating behaviour of females from the previously studied species and consequent sperm competition may contribute to the rapid gene evolution in its male-biased genes, sperm competition is unlikely to contribute to the rapid evolution of male-biased genes in the female monogamous *A. gambiae*. Accordingly, the male-biased cluster M3 that mainly contains genes expressed in the testis and sperm do not show any sign of rapid evolution. Accessory gland genes have been also reported to evolve rapidly in insects, [29], [30]; however, only 8 of the known *A. gambiae* accessory gland genes [31], [32] were represented in the male-biased set of this study. It is therefore reasonable to exclude the possibility that these genes have any significant influence in the observed differences of unique sequences and the codon usage of the male-biased genes. A consequence of the monogamous behaviour of *A. gambiae* females is that a single male mosquito fertilizes all the eggs produced by a female during her lifetime. Clearly this exerts a strong selection pressure, at the level of pre-copulation processes such as swarming, on those genes that increase the mating success of *A. gambiae* males. In particular, the ability of male mosquitoes to form swarms seems crucial to effectively attract females [33]. This is a complex and high energy consuming behavioural process that favours traits increasing male fitness and survival as well as male:male competition, and could therefore exert a powerful evolutionary pressure on male-specific genes.

Cross-species comparison of the transcriptional profiles from *A. gambiae* sex-biased genes with orthologues in *D. melanogaster* reveals that very few male-biased genes have conserved expression profiles while female-biased and non-sex-biased genes show significantly less transcriptional variance. Such a high degree of conservation in female-biased genes is remarkable considering that several *A. gambiae* female-biased genes must be involved in processes that are not shared with the female fruit fly, such as host odour recognition and blood feeding. Accordingly, our data show a significant increase in the codon usage bias of *A. gambiae* female-biased genes as compared to non-sex-biased genes. All together these findings indicate that a subset of adult male- and female-biased genes evolve in opposite directions. In early developmental stages, female-biased genes transcribed at the pupal stage show significantly less *D. melanogaster* orthologues than the rest of the genome thus providing clues for the presence of different evolutionary pressures acting upon developmentally regulated female-biased genes. Consistent with our hypothesis that evolutionary pressures act on processes involved in adult fitness rather than the sperm, male-biased genes transcribed at early stages do not show signs of rapid evolution.

The concerted expression of a large number of known *Anopheles* immunity-related genes in female pupae could be the result of a systematic activation of the immune system elicited by the extensive process of larval tissue histolysis that accompanies the formation of adult female organs and tissues during pupal development. Possibly differences in timing of organ histolysis between males and females could explain the female-biased expression of the immunity genes. This hypothesis is in agreement with the observation that the histolysis of larval organs during *D. melanogaster* metamorphosis induced an immune response in early pupae [34]. However, immunity genes were not found to be female-biased during *D. melanogaster* pupation [35] indicating that the comparatively strong activation of the *A. gambiae* female immune response is not observed in all insects.

We have identified 26 novel testis-specific genes and 2 novel ovary-specific genes as well as 4 genes transcribed specifically in both the male and female gonads. This information provides a significant addition to our understanding of the transcriptional programmes leading to gonad development and gamete differentiation. Our findings demonstrate, for the first time in *Anopheles*, the possibility of carrying out functional studies on organ development by employing embryonic RNAi. A fraction of the mosquito injected with a dsRNA targeting AGAP006449, a gene that encodes a protein showing significant similarities to *SDS3* showed a complete developmental arrest of the gonad, irrespectively of the sex. This is a phenotype very similar to that observed in *D. melanogaster* carrying mutations in the sequence of the *zpg* innexin 4, gene [27]. Accordingly the injection of dsRNA targeting AGAP006241 the putative innexin orthologue in *A. gambiae* impaired gonad development in a substantial fraction of injected mosquitoes. In several species it has been shown that SDS3 increases the enzymatic activity of HDAC1 that functions as transcription repressor by remodeling the chromatin structure at specific sites where it is selectively recruited by sequence-specific DNA-binding proteins [36]. AGAP006449 shares sequence homology to the *D. melanogaster* gene CG14220. The biological processes in which CG14220 is involved are not known and no phenotypic data are available. Our findings would suggest that AGAP006449 ought to play a crucial role in the early stages of gonad development in both male and female mosquitoes.

While the identification and the functional characterization of novel of gonadal genes will help in the design of genetic vector control measures targeting the reproduction capability of the mosquito population, the availability of adult female mosquitoes lacking both gonads through embryo injections will prove invaluable to elucidate the function of the ovaries in regulating the blood meal dependent induction of gut and the fat body genes as well as in unravelling the role of the ovaries in modulating the insect immune response. Further investigations of interfering with gonad-specific gene expression may result in more efficient ways of generating mosquitoes lacking gonads.

## Materials and Methods

### Microarray hybridizations and data analysis

The Anopheles MMC2 microarray platform was used for all hybridizations presented in this study, allowing evaluation of 7246 *A. gambiae* genes (AgamP3.3 March 2008). Total RNA pools were obtained from sexed *A. gambiae* (strain G3) *dsx*-EGFP larvae, pupae and adults (fed on 10% glucose (vw/v) solution) by TRIZOL® extraction (Invitrogen) on three biological replicates for each of the five developmental time point as shown in Figure S1 and processed for microarray hybridizations as previously described [37]. Data was acquired and filtered as described by Vlachou et al. [38] and subsequently uploaded to GeneSpring GX 7.3 software package (Agilent technologies) for normalization by the locally weighted linear regression (Lowess) method. Genes exhibiting expression values in at least 66% of all biological replicates were subjected to a one-way ANOVA statistical test (P≤0.05) based on errors calculated by a cross-gene error model. A small fraction of genes that failed to pass the statistical filter was recovered for further analysis if the average of all biological replicates showed an expression ratio superior to 0.6 log_2_ and at least 66% of the biological replicates showed the same direction of regulation. In total, this approach yielded 6142 genes with high confidence expression data for at least one developmental time point. Genes were regarded as significantly female and male-biased when showing male-female expression ratios lower or higher than a 1.73-folds (log_2_0.8) cut-off respectively, as determined previously by self-hybridization. This yielded a total of 1884 sex-biased genes across all time points examined. All microarray data is MIAME compliant and the raw data has been deposited in the ArrayExpress database (accession number E-MEXP-3093). The 454 sequencing analysis method can be found in Methods S1.

### Comparative genomics and transcriptomics

Orthology was assessed using the Ensembl Anopheles genome release V53 (March 2009) with respect to the *Ae. aegypti* and *D. melanogaster* data set. Statistically significant differences in the frequency of orthologues were detected by hypergeometric distribution as calculated with Excel (Microsoft Corp., Redlands, WA) and corrected according to the Bonferroni method. Several sequence quality control steps were taken for codon usage bias analysis, as previously suggested [25], including: presence of an ATG start codon, sequence length being a multiple of three, absence of internal stop codons and selection of only the longest transcript in the case of genes with multiple transcripts. This analysis yielded a collection of 4970 sequences for which the effective number of codons (ENC) [39] and the frequency of optimal codons (Fop) [40] was calculated using the CodonW program (http://bioweb.pasteur.fr/seqanal/interfaces/codonw.html) and statistically using the nonparametric Kolmogorov-Smirnov test. Optimal codons were established by scoring all the available sequences for analysis. According to the ENC index, lower values than the reference set indicate stronger codon usage bias associated to slower evolutionary rates while higher values indicate weaker codon usage bias and selection intensity. Data of *Drosophila melanogaster* sex-biased expression was obtained from the Sebida database (http://141.61.102.16:8080/sebida/index.php) based on previously published data [6] and a non redundant set of one to one orthologues with *A. gambiae* genes was established for specifically for this analysis.

### Expression clustering and compositional analysis

QT clustering was performed at various thresholds with the GeneSpring GX 7.3 software package (Agilent technologies) to identify the most suitable number of cluster to use for K-means clustering analysis which was performed using the Cluster 3.0 software (http://bonsai.hgc.jp/~mdehoon/software/cluster/) for all sex-biased genes whose expression profiles contained values for at least 3 of the 5 developmental stages. Expression clusters were visualized with the Java Treeview 1.0.8 software (http://genetics.stanford.edu/~alok/TreeView/). Clustering of testis- and germline-specific genes was done with the Cluster 3.0 software and a weighted hierarchical method. The frequency of GO terms and InterPro domains was analysed using the GeneMerge software by comparing the genes within the k-means clusters to all 6142 genes analysed.

### Transcriptional Profiling

Total RNA was prepared using the TRI reagent (Ambion). Following RQ1 DNAse (Promega) treatment. cDNA was made using Superscript II (Invitrogen) and Oligo dt Primers (Invitogen) following the manufacturer's instructions. RT-PCR experiments were performed using the Hotstar Plus polymerase (Qiagen). The primers used to identify the testis, the ovary and the gonad-specific genes can be found in Table S12. The RT-PCR results for all genes tested for tissue-specificity is provided in the supplementary material (Tables S5 and S6). The primers for all genes used for tissue-specific RT-PCRs will be provided upon request. Quantitative real-time PCRs (qRT-PCR) were performed on cDNA using the Fast SYBR-Green master mix and ABI PRISM 7000 Sequence Detector (Applied Biosystems). In order to normalize the amount of RNA in each reaction internal controls using the *A. gambiae* RPL19 ribosomal gene were performed. Three independent biological replicates were subjected to duplicate technical assays. qRT-PCR primers can be found in Table S12.

### Functional analysis

dsRNA for embryo injections was generated using the T7 megascript kit (Ambion) following the manufacturer's instructions using PCR fragments generated with T7 primers as a template. The primers utilized can be found in Table S12. The dsRNA was suspended in injection buffer (50 mM KCl, 1 mM NaPO_4_, pH7.2) at a concentration of 1 µg/µL. Approximately 0.1 ng dsRNA was injected into preblastodermic embryos as described below for transgenesis with the modification of inserting the needle in the embryos at the posterior 1/3 of their length on the ventral side.

### Plasmid construction and development of transgenic line

To generate the construct pPB(3xP3-DsRed)β2–eGFP, Act5C *dsx*–eGFP plasmid (Figure S1), 2.5 kb of the *A. gambiae dsx* gene from exon 4 until 6 was amplified by PCR from *A. gambiae* (strain G3) genomic DNA using the primers BamHI dsx F CCC GGA TCC GCC ATC TTC ATT GTT TTG TCG TGA AGA GCG CCG ATG GCG, XbaI dsx R CCC TCT AGA GTC AGA TAC ATC ACG ATT GCC ACC GAG ATG TTC TCG TCC. The *dsx* fragment was inserted into the pSL-Act plasmid downstream of the *Drosophila Actin*5C promoter and upstream of the *Drosophila hsp*70 terminator [41]. The eGFP sequence was inserted in-frame into the HincII restriction site at the beginning of the 5^th^ exon of *dsx*. The 3.2 kb Act *dsx* eGFP *hspT* fragment was inserted into the transformation plasmid pPB(3xP3-DsRed) using the AscI restriction site. The β2-eGFP fragment [42] was cloned into the transformation plasmid to create pPB{3xP3-DsRed}β2–eGFP, Act5C *dsx*–eGFP. The plasmid was co-injected into *A. gambiae* (strain G3) embryos with a helper plasmid containing the *piggyBac* transposase as previously described [43], [44]. Following the injection of 1200 embryos the surviving 71 adults (5.9%) were crossed to wild-type mosquitoes. The progeny of the crosses were screened for 3xP3 DsRed expression. Two transgenic larvae were identified whereof one female survived to adulthood. The *dsx* transgenic line was established from this single female founder. inverse PCR analysis showed that the pPB[DsRed]β2–eGFP, Act5C *dsx*–eGFP cassette integrated into a single location, within the fourth intron of AGAP006528 on chromosome 2L.

## Supporting Information

Figure S1 **Development and phenotypic characterization of** ***dsx*** **–eGFP mosquitoes.** The transgenic mosquitoes dsx-eGFP were developed by injecting the transformation construct pPB[DsRed]β2–EGFP-Act5C *dsx*–eGFP together with a source of transposase into preblastodermic embryos. The constructs contains three transcription units flanked by the *piggyBac* inverted repeats (pBL and pBR) including from 5′ to 3′: 1) the 3xP3 neural-ganglia-specific promoter, the DsRed sequence and SV 40 terminator, 2) The testis-specific β2-tubulin promoter, the eGFP coding sequence and the 3′ β2-tubulin untranslated region; the actin 5C promoter, the eGFP coding sequence engineered to contain at its 5′ and 3′ end intron 4 and 5 of the *A. gambiae* sex differentially spliced gene doublesex (*dsx*) and the *D. melanogaster* Hsp terminator. (B) Transmission and green and red fluorescence overlay micrographs of 1 day old mosquito larvae. Male and female larvae can be easily and reliably distinguished on the basis of differential eGFP transcription of the actin 5C promoter as early as after hatching. While male larvae show a strong eGFP expression, this marker is almost undetectable in female individuals. inverse PCR analysis showed that the pPB[DsRed]β2–eGFP, Act5C *dsx*–eGFP cassette integrated into a single location, within the fourth intron of AGAP006528 on chromosome 2L. The *Drosophila* orthologue of AGAP006528 is involved in compound eye photoreceptor development and is therefore not likely responsible for the sex-biased expression of eGFP. No abnormal eye, or other, phenotypes have been observed in the *dsx* transgenic line (data not shown). (C) Male and female dsx-eGFP mosquitoes collected at different developmental stages were separated using either the fluorescent visible markers (from larvae to pupae) or phenotypic traits (adults) and utilized to prepare differentially labelled microarray hybridization probes. The life stages examined included 1^st^ instar (L1) 2^nd^ and 3^rd^ instar larvae (L2 and L3), 4^th^ instar larvae (L4), pupae and three-day-old virgin non-blood-fed adults.
(PDF)

Figure S2 **Comparison of microarray transcription analysis and 454 sequencing of larval stages RNA.** The raw fluorescence signal obtained from the hybridization of 1^st^ instar male (A) and female (B), onto the MMC2 microarray was compared with the number of transcript traces generated by 454 sequencing of the same mRNA starting material. The total number of genes (N) analyzed to calculate the Spearman correlation coefficient is present on top. Each point represents an individual gene.
(PDF)

Figure S3 **Comparison of micro-array and qRT–PCR transcription analysis.** At different developmental stages we compared the male:female expression ratios deduced from micro-array hybridization data (red) with the values obtained by qPCR analysis (blue). For this analysis we selected three genes with distinct developmental expression patterns: (A) AGAP003087 a female-biased ovary specific gene; (B) AGAP006241 a female biased gene; and (C) AGAP001416 a male-biased testis-specific gene.
(PDF)

Figure S4 **Comparison of adult microarray transcription and previously available microarray data.** Male:female expression ratios for adult *A. gambiae* mosquitoes were compared to previously available microarray data for the same developmental stage. The total number of genes (N) analyzed to calculate the Spearman correlation coefficient is present on top. Each point represents an individual gene.
(PDF)

Figure S5 **Relationship between codon bias and the degree of sex-biased expression.** The frequency of optimal codons (Fop) is plotted for 503 male-biased (blue) against the male/female expression ration (Spearman rank correlation, R =  −0.14, P = 0.001) and for 954 female-biased (pink) genes against female/male expression ratio (Spearman rank correlation, R =  0.068, P = 0.037).
(PDF)

Figure S6 **Phylogenomic analysis of co-expressed genes.** Male-biased (blue) and female-biased (pink) genes of M1-M4 and F1-F4 clusters were analysed for the percentage of unique sequences by comparing the genomes of *A. gambiae* with *D. melanogaster* (An:Dm), *A. gambiae* with *Ae. Aegypti* (An:Ae) and *A. gambiae* with *Ae agypti* and *D. melanogaster* (An:Ae:Dm). Differences in the percentage of unique sequences compared to that observed in the subset of genes of male- and female-biased clusters (grey) were statistically evaluated using Bonferroni corrected hypergeometric distribution (P<0.05, two asterisks).
(PDF)

Figure S7 **Validation of Embryonic RNAi Technique in** ***A. gambiae.*** Experiments validating the efficiency of embryonic RNAi were performed by targeting the DsRed transgenic marker gene driven by the eye- and neural-ganglia-specific 3xP3 promoter. This promoter drives expression from the late embryonic stages. RNAi efficiency was estimated visually. (A) dsRNA control injections targeting the bacterial β-galactosidase gene (*lacZ*) showed no reduction in DsRed expression compared with that in non-injected individuals. However, approximately 50% of the surviving DsRed-dsRNA-injected mosquitoes showed either partial (B) or a complete (C) absence of DsRed fluorescence, which lasted throughout the larval stages. The partial knock-down phenotypes lacked DsRed fluorescence in the posterior end of the larvae, whereas they displayed weak to normal anterior DsRed fluorescence (B). The DsRed-negative larvae frequently exhibited a return of fluorescence in the eyes at the pupal stage.
(PDF)

Figure S8 **qRT-PCR analysis of dsRNA injected mosquitoes.** Knock down efficiency following embryonic RNAi was analysed in adult mosquitoes. The data was normalized to *lacZ* control values of 100% (A) The bars show the relative transcript levels of AGAP006449 detected in mosquitoes injected with *lacZ* dsRNA and AGAP006449 dsRNA. (B) Relative AGAP006421 transcript levels detected in *lacZ* dsRNA and AGAP006241 dsRNA injected mosquitoes.
(PDF)

Table S1 **List of all** ***A. gambiae*** **genes with expression values over the background.**
(XLS)

Table S2 **List of all** ***A. gambiae*** **genes showing a sex-biased transcription at one or more of the developmental stages examined.**
(XLS)

Table S3 **List of genes showing a sex-bias transcription profile from the late larval stage to adulthood.**
(PDF)

Table S4 **List of genes contained in each cluster.**
(XLS)

Table S5 **List of male-biased genes tested for tissue specific RT-PCR (91 genes).**
(XLS)

Table S6 **List of female-biased genes tested for tissue specific RT-PCR (76 genes).**
(XLS)

Table S7 **Distribution of GO ID terms amongst the genes of the K-means clusters.**
(PDF)

Table S8 **Distribution of InterPro domains amongst the genes of the K-means clusters.**
(PDF)

Table S9 **Observed and expected frequency of unique sex-biased genes in** ***A. gambiae.***
(PDF)

Table S10 **Observed and expected frequency of unique sex biased genes within individual K-means clusters.**
(PDF)

Table S11 **List of genes with a conserved male-biased transcription profile in adult** ***A. gambiae*** **and** ***D. melanogaster.***
(PDF)

Table S12 **List of oligonucleotide primers utilized in the amplification experiments.**
(XLS)

Methods S1 (DOC)
