# Supplementary figures and images for: Transcription Regulation of Sex-Biased Genes during Ontogeny in the Malaria Vector Anopheles gambiae
Source: PLoS One. 2011 Jun 30;6(6):e21572. doi: 10.1371/journal.pone.0021572 (PMC3128074; doi:10.1371/journal.pone.0021572)

**Figure S1**

**A**

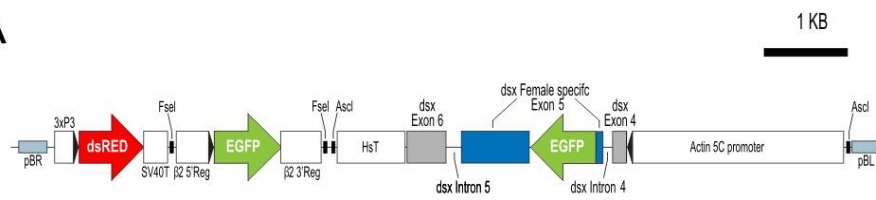

**B**

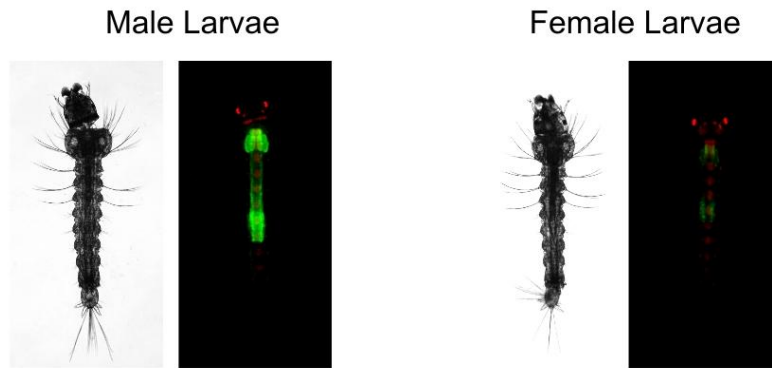

**C**

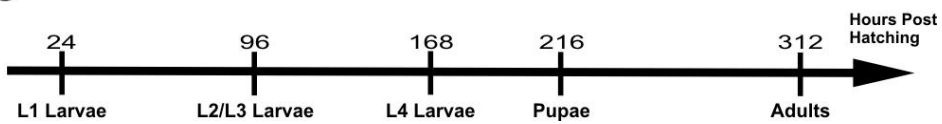

Supplement: Figure S1 — Development and phenotypic characterization of dsx –eGFP mosquitoes. The transgenic mosquitoes dsx-eGFP were developed by injecting the transformation construct pPB[DsRed]β2–EGFP-Act5C dsx–eGFP together with a source of transposase into preblastodermic embryos. The constructs contains three transcription units flanked by the piggyBac inverted repeats (pBL and pBR) including from 5′ to 3′: 1) the 3xP3 neural-ganglia-specific promoter, the DsRed sequence and SV 40 terminator, 2) The testis-specific β2-tubulin promoter, the eGFP coding sequence and the 3′ β2-tubulin untranslated region; the actin 5C promoter, the eGFP coding sequence engineered to contain at its 5′ and 3′ end intron 4 and 5 of the A. gambiae sex differentially spliced gene doublesex (dsx) and the D. melanogaster Hsp terminator. (B) Transmission and green and red fluorescence overlay micrographs of 1 day old mosquito larvae. Male and female larvae can be easily and reliably distinguished on the basis of differential eGFP transcription of the actin 5C promoter as early as after hatching. While male larvae show a strong eGFP expression, this marker is almost undetectable in female individuals. inverse PCR analysis showed that the pPB[DsRed]β2–eGFP, Act5C dsx–eGFP cassette integrated into a single location, within the fourth intron of AGAP006528 on chromosome 2L. The Drosophila orthologue of AGAP006528 is involved in compound eye photoreceptor development and is therefore not likely responsible for the sex-biased expression of eGFP. No abnormal eye, or other, phenotypes have been observed in the dsx transgenic line (data not shown). (C) Male and female dsx-eGFP mosquitoes collected at different developmental stages were separated using either the fluorescent visible markers (from larvae to pupae) or phenotypic traits (adults) and utilized to prepare differentially labelled microarray hybridization probes. The life stages examined included 1st instar (L1) 2nd and 3rd instar larvae (L2 and L3), 4th instar la [file pone.0021572.s001.pdf]

**Figure S2**

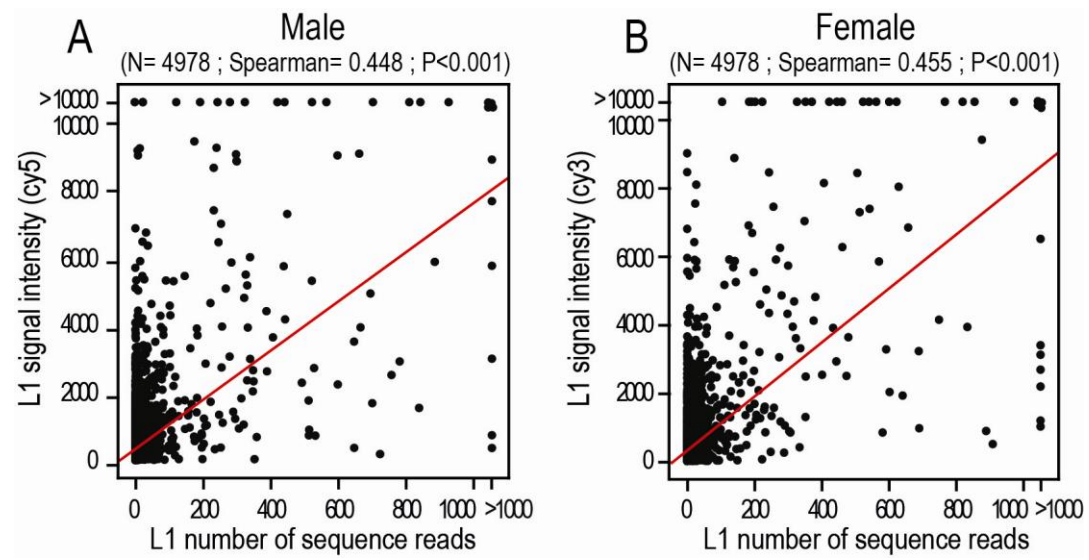

Supplement: Figure S2 — Comparison of microarray transcription analysis and 454 sequencing of larval stages RNA. The raw fluorescence signal obtained from the hybridization of 1st instar male (A) and female (B), onto the MMC2 microarray was compared with the number of transcript traces generated by 454 sequencing of the same mRNA starting material. The total number of genes (N) analyzed to calculate the Spearman correlation coefficient is present on top. Each point represents an individual gene. (PDF) [file pone.0021572.s002.pdf]

Figure S3

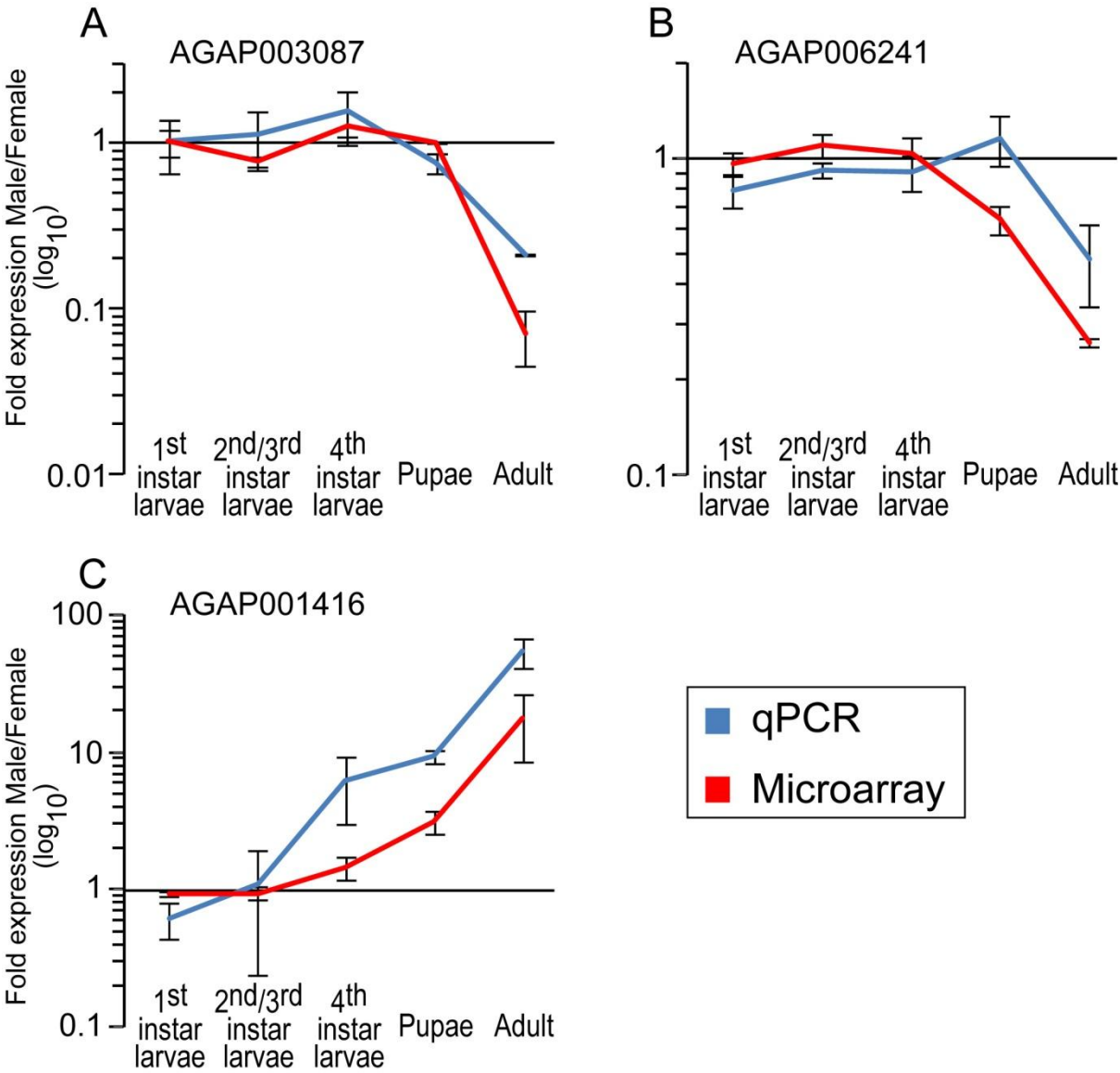

Supplement: Figure S3 — Comparison of micro-array and qRT–PCR transcription analysis. At different developmental stages we compared the male:female expression ratios deduced from micro-array hybridization data (red) with the values obtained by qPCR analysis (blue). For this analysis we selected three genes with distinct developmental expression patterns: (A) AGAP003087 a female-biased ovary specific gene; (B) AGAP006241 a female biased gene; and (C) AGAP001416 a male-biased testis-specific gene. (PDF) [file pone.0021572.s003.pdf]

Figure S4

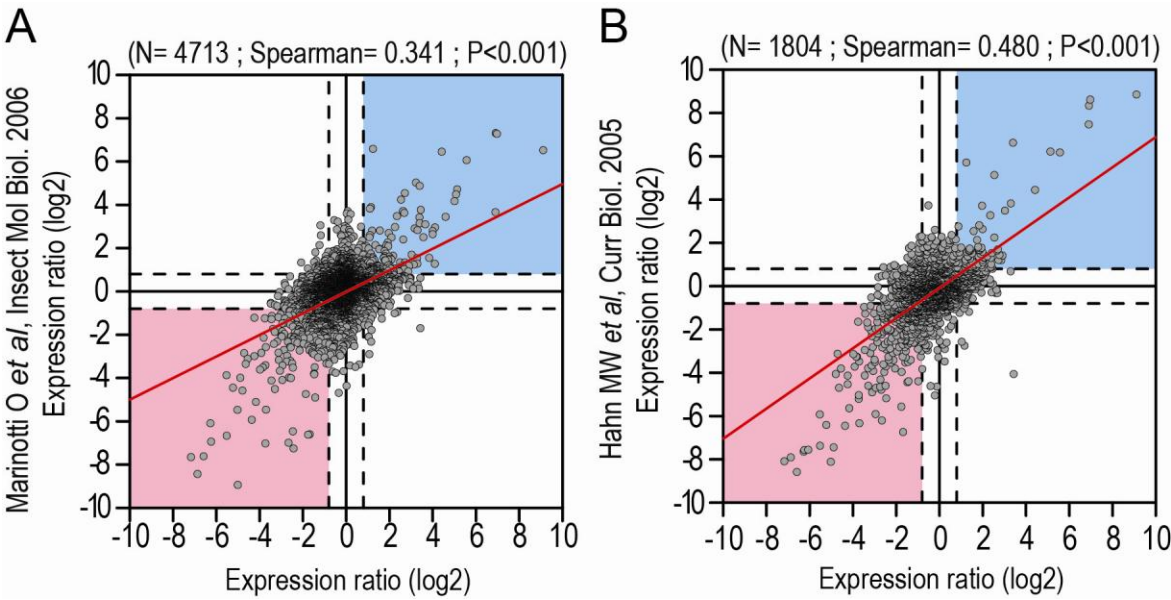

Supplement: Figure S4 — Comparison of adult microarray transcription and previously available microarray data. Male:female expression ratios for adult A. gambiae mosquitoes were compared to previously available microarray data for the same developmental stage. The total number of genes (N) analyzed to calculate the Spearman correlation coefficient is present on top. Each point represents an individual gene. (PDF) [file pone.0021572.s004.pdf]

**Figure S5**

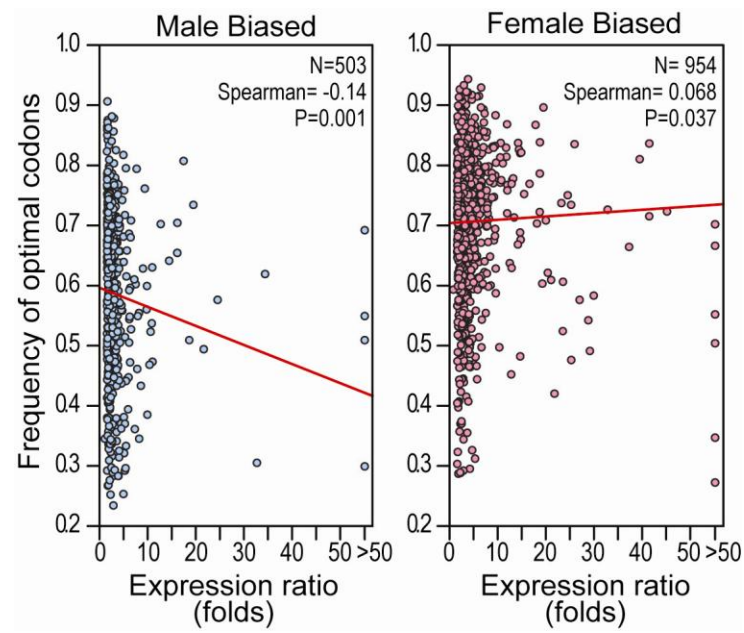

Supplement: Figure S5 — Relationship between codon bias and the degree of sex-biased expression. The frequency of optimal codons (Fop) is plotted for 503 male-biased (blue) against the male/female expression ration (Spearman rank correlation, R = −0.14, P = 0.001) and for 954 female-biased (pink) genes against female/male expression ratio (Spearman rank correlation, R = 0.068, P = 0.037). (PDF) [file pone.0021572.s005.pdf]

Figure S6

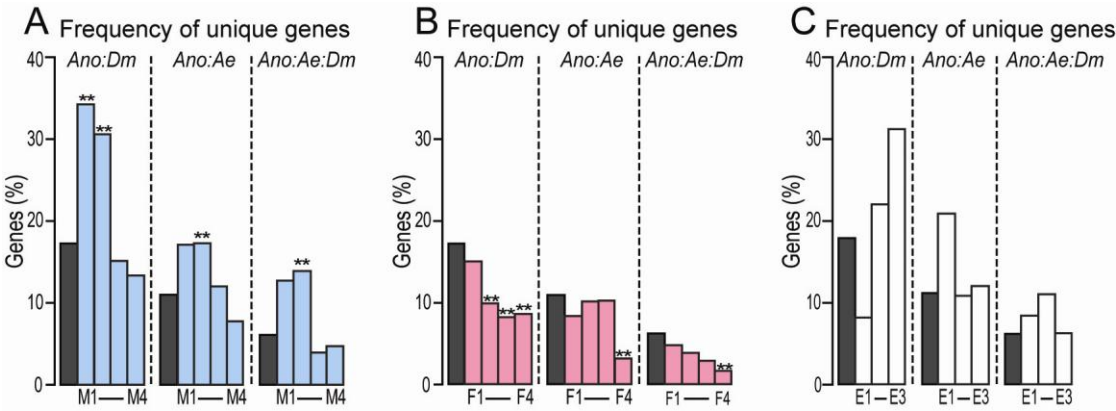

Supplement: Figure S6 — Phylogenomic analysis of co-expressed genes. Male-biased (blue) and female-biased (pink) genes of M1-M4 and F1-F4 clusters were analysed for the percentage of unique sequences by comparing the genomes of A. gambiae with D. melanogaster (An:Dm), A. gambiae with Ae. Aegypti (An:Ae) and A. gambiae with Ae agypti and D. melanogaster (An:Ae:Dm). Differences in the percentage of unique sequences compared to that observed in the subset of genes of male- and female-biased clusters (grey) were statistically evaluated using Bonferroni corrected hypergeometric distribution (P<0.05, two asterisks). (PDF) [file pone.0021572.s006.pdf]

**Figure S7**

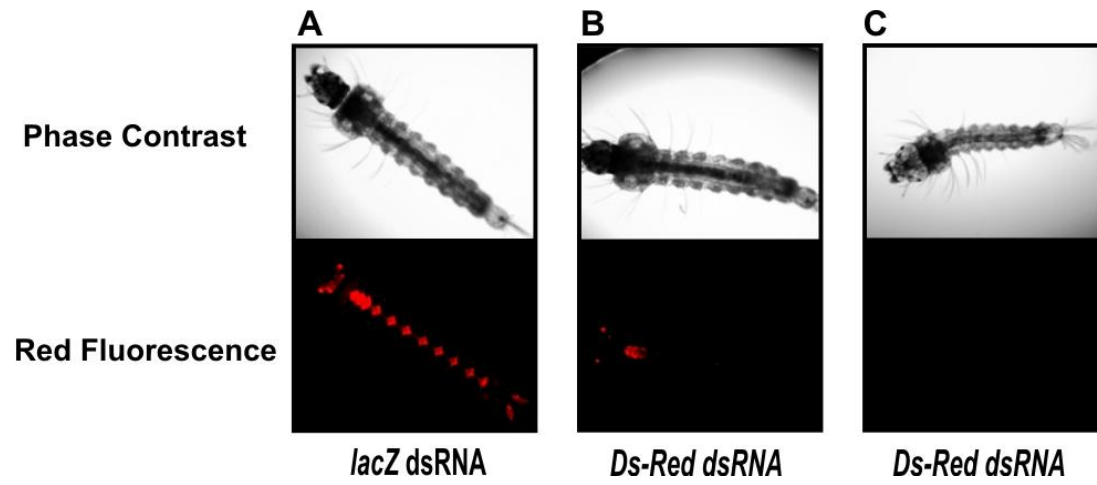

Supplement: Figure S7 — Validation of Embryonic RNAi Technique in A. gambiae. Experiments validating the efficiency of embryonic RNAi were performed by targeting the DsRed transgenic marker gene driven by the eye- and neural-ganglia-specific 3xP3 promoter. This promoter drives expression from the late embryonic stages. RNAi efficiency was estimated visually. (A) dsRNA control injections targeting the bacterial β-galactosidase gene (lacZ) showed no reduction in DsRed expression compared with that in non-injected individuals. However, approximately 50% of the surviving DsRed-dsRNA-injected mosquitoes showed either partial (B) or a complete (C) absence of DsRed fluorescence, which lasted throughout the larval stages. The partial knock-down phenotypes lacked DsRed fluorescence in the posterior end of the larvae, whereas they displayed weak to normal anterior DsRed fluorescence (B). The DsRed-negative larvae frequently exhibited a return of fluorescence in the eyes at the pupal stage. (PDF) [file pone.0021572.s007.pdf]

Figure S8

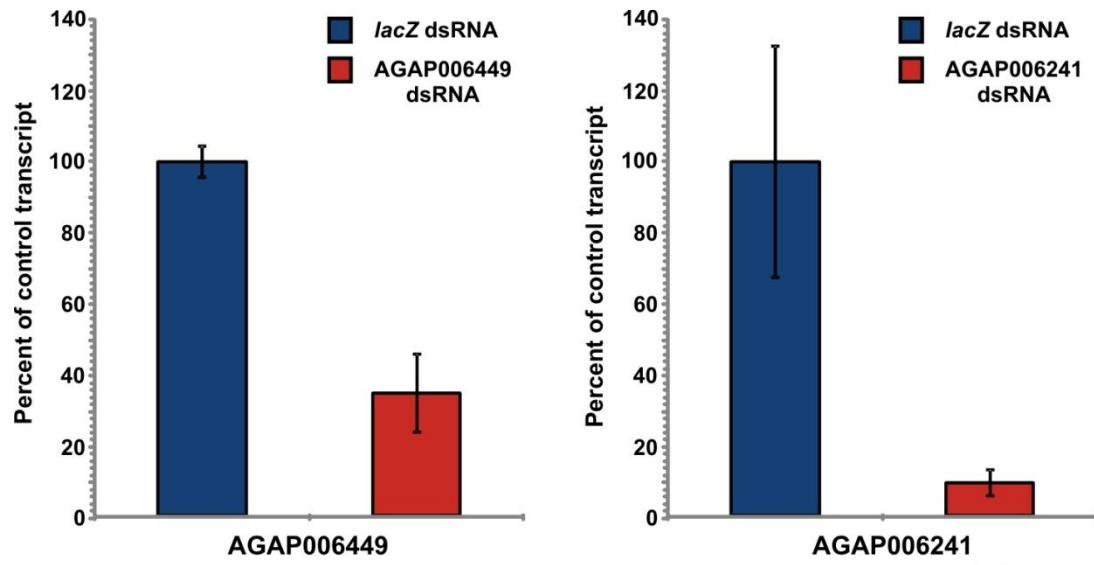

Supplement: Figure S8 — qRT-PCR analysis of dsRNA injected mosquitoes. Knock down efficiency following embryonic RNAi was analysed in adult mosquitoes. The data was normalized to lacZ control values of 100% (A) The bars show the relative transcript levels of AGAP006449 detected in mosquitoes injected with lacZ dsRNA and AGAP006449 dsRNA. (B) Relative AGAP006421 transcript levels detected in lacZ dsRNA and AGAP006241 dsRNA injected mosquitoes. (PDF) [file pone.0021572.s008.pdf]
