# Supplementary material for: Transcription Regulation of Sex-Biased Genes during Ontogeny in the Malaria Vector Anopheles gambiae
Source: PLoS One. 2011 Jun 30;6(6):e21572. doi: 10.1371/journal.pone.0021572 (PMC3128074; doi:10.1371/journal.pone.0021572)
Supplement: Table S3 — List of genes showing a sex-bias transcription profile from the late larval stage to adulthood. (PDF) [file pone.0021572.s011.pdf]

Table S3

List of genes showing a sex-bias transcription profile from the late larval stage to adulthood

	Gene ID	Interpro domain ID & Description	Male:female Expression Ratio (Log_2) 4th instar larvae	Pupae	Adult mosquitoes
**Male-Biased**	AGAP001388	IPR001275: DM DNA-binding	1.95	1.43	2.12
	AGAP001620		3.51	4.3	5.11
	AGAP001735	IPR008805: RIB43A	1.38	1.67	1.83
	AGAP002033	IPR001589: Actin-binding, actinin-type	3.47	3.61	3.43
	AGAP002365		2.89	3.31	1.89
	AGAP002515	IPR008271: Serine/threonine protein kinase, active site	1.02	1.01	3.42
	AGAP003022		2.55	3.09	4.03
	AGAP003747		3.65	2.76	5.6
	AGAP003895	IPR006651: Kelch motif	1.4	1.41	1.95
	AGAP004862	IPR001565: Synaptotagmin	2.95	2.45	2.68
	AGAP004914	IPR000217: Tubulin	2.93	3.55	4.63
	AGAP005179	IPR005334: Tctex-1	2.49	2.91	4.3
	AGAP005748	IPR007087: Zinc finger, C2H2-type	1.1	1.31	1.61
	AGAP005850		0.88	1.30	1.67
	AGAP006622	IPR003299: Flagellar calcium-binding protein (calflagin)	2.14	2.17	3.69
	AGAP006706		2.28	1.45	2.29
	AGAP006887	IPR001372: Dynein light chain, type 1	2.15	1.9	3.87
	AGAP007664	IPR000956: Stathmin	4.20	3.14	4.45
	AGAP007904	IPR000819: Peptidase M17, leucyl aminopeptidase	2.38	2.9	2.57
	AGAP008186	IPR000533: Tropomyosin	1.15	1.31	2.5
	AGAP008338	IPR007914: Protein of unknown function UPF0193	1.28	1.22	2.9
	AGAP008341	IPR008271: Serine/threonine protein kinase, active site	1.04	1.7	2.55
	AGAP008765	IPR008271: Serine/threonine protein kinase, active site	2.5	2.25	3.26
	AGAP008850	IPR001349: Cytochrome c oxidase, subunit Via	2.72	1.04	4.03
	AGAP008976	IPR002373: cAMP/cGMP-dependent protein kinase	2.69	2.58	1.59
	AGAP009026	IPR006553: Leucine-rich repeat, cysteine-containing subtype	2.56	1.89	2.74
	AGAP010031	IPR000435: Tektin	2.49	2.32	2.72
	AGAP010199	IPR003409: MORN motif	3.21	2.77	5.03
	AGAP010330		2.35	2.4	5.04
	AGAP010341	IPR008271: Serine/threonine protein kinase, active site	1.54	0.97	3.37
	AGAP011102		2.15	2.63	3.2
	AGAP012224	IPR007701: Interferon-related developmental regulator	2.78	3.58	4.13
	AGAP012739		0.89	1.01	1.71
**Bimodal sex bias***	AGAP011630		-0.82	-1.08	1.62
**Female-Biased**	AGAP001182	IPR001187: Tissue factor	-0.8	-0.98	-2.92
	AGAP001621	IPR001608: Alanine racemase	-1.7	-1.52	-1.32

*This gene is female-biased in 4th instar larvae but becomes male-biased at the pupal and adult stages
