# Supplementary material for: Transcription Regulation of Sex-Biased Genes during Ontogeny in the Malaria Vector Anopheles gambiae
Source: PLoS One. 2011 Jun 30;6(6):e21572. doi: 10.1371/journal.pone.0021572 (PMC3128074; doi:10.1371/journal.pone.0021572)
Supplement: Table S7 — Distribution of GO ID terms amongst the genes of the K-means clusters. (PDF) [file pone.0021572.s015.pdf]

Table S7

Distribution of GO ID terms amongst the genes of the K-means clusters

Cluster [genes]	GO Class	GO ID	GO Description	Freq. in total [6142]	Occur. in total [6142]	Freq. in cluster	Occur. in cluster	P-value
M1 [96]	Mol. Func.	GO:05529	sugar binding	4.1E-03	25	0.04	4	5.2E-04 *
	Mol. Func	GO:08080	N-acetyltransferase activity	2.8E-03	17	0.03	3	0.002*
	Mol. Func	GO:16651	oxidoreductase activity	9.8E-04	6	0.02	2	0.003*
	Bio. Proc	GO:15031	protein transport	5.2E-03	32	0.03	3	0.012
M2 [333]	Mol. Func	GO:04089	carbonate dehydratase activity	1.3E-03	8	0.01	3	0.007*
	Bio. Proc.	GO:06730	carbon compound metabolic proc.	1.5E-03	9	0.01	3	0.010*
	Cel. Comp.	GO:05576	extracellular region	9.4E-03	58	0.02	8	0.009
	Mol. Func	GO:04785	superoxide dismutase activity	6.5E-04	4	0.01	2	0.016*
M3 [91]	Cel. Comp.	GO:05874	Microtubule	3.9E-03	24	0.05	5	2.1E-05 *
	Bio. Proc.	GO:00226	microtubule cytoskeleton org.	2.0E-03	12	0.03	3	6.1E-04*
	Bio. Proc.	GO:07017	microtubule-based process	1.6E-03	10	0.02	2	0.009*
	Mol. Func	GO:03777	microtubule motor activity	3.4E-03	21	0.02	2	0.035
M4 [92]	Cel. Comp.	GO:16469	two-sector ATPase complex	2.9E-03	18	0.02	2	0.027
	Mol. Func	GO:46961	proton-transporting ATPase activity	3.1E-03	19	0.02	2	0.030
	Mol. Func	GO:46933	proton-transporting ATP synthase	3.1E-03	19	0.02	2	0.030
	Bio. Proc.	GO:15986	ATP synt. coupled proton transport	3.1E-03	19	0.02	2	0.030
F1 [156]	Cel. Comp.	GO:05840	Ribosome	1.1E-02	69	0.10	16	7.7E-12 *
	Mol. Func	GO:03735	structural constituent of ribosome	1.2E-02	72	0.10	16	1.5E-11 *
	Bio. Proc.	GO:06412	Translation	1.6E-02	100	0.10	16	2.6E-09 *
	Cel. Comp.	GO:30529	ribonucleoprotein complex	8.1E-03	50	0.06	10	3.3E-07 *
F2 [726]	Bio. Proc.	GO:06412	Translation	1.6E-02	100	0.05	38	7.9E-12 *
	Mol. Func	GO:05524	ATP binding	6.2E-02	381	0.12	87	1.7E-10 *
	Cel. Comp.	GO:05840	Ribosome	1.1E-02	69	0.03	24	3.7E-07 *
	Mol. Func	GO:04386	helicase activity	8.8E-03	54	0.03	20	1.1E-06 *
F3 [88]	Bio. Proc.	GO:07010	cytoskeleton organization	9.8E-04	6	0.02	2	0.003*
	Cel. Comp.	GO:00785	Chromatin	2.6E-03	16	0.02	2	0.020*
	Bio. Proc.	GO:06911	phagocytosis, engulfment	0.01	78	0.05	4	0.020
	Bio. Proc.	GO:07242	Intracellular signalling cascade	8.1E-03	50	0.03	3	0.029
F4 [151]	Mol. Func	GO:04813	tRNA ligase activity	3.3E-04	2	0.01	2	6.0E-04*
	Bio. Proc.	GO:06419	tRNA aminoacylation	3.3E-04	2	0.01	2	6.0E-04*
	Bio. Proc.	GO:06094	gluconeogenesis	4.9E-04	3	0.01	2	0.002*
	Mol. Func	GO:17111	nucleoside-triphosphatase activity	9.0E-03	55	0.04	6	0.002*
E1 [14]	Mol. Func	GO:16491	oxidoreductase activity	3.2E-02	196	0.21	3	0.008*
E2 [9]			No over- represented GO terms					
E3 [34]	Mol. Func	GO:04866	endopeptidase inhibitor activity	2.0E-03	12	0.18	6	1.6E-11 *
	Bio. Proc.	GO:42742	defense response to bacterium	6.5E-04	4	0.09	3	6.2E-07 *
	Bio. Proc.	GO:45087	innate immune response	1.1E-03	7	0.09	3	5.3E-06 *
	Bio. Proc.	GO:06955	immune response	1.1E-03	7	0.09	3	5.3E-06 *

* Statistically significant overrepresentation according to Bonferroni corrected hypergeometric distribution.
