# Supplementary material for: Transcription Regulation of Sex-Biased Genes during Ontogeny in the Malaria Vector Anopheles gambiae
Source: PLoS One. 2011 Jun 30;6(6):e21572. doi: 10.1371/journal.pone.0021572 (PMC3128074; doi:10.1371/journal.pone.0021572)
Supplement: Table S8 — Distribution of InterPro domains amongst the genes of the K-means clusters. (PDF) [file pone.0021572.s016.pdf]

Table S8

Distribution of InterPro domains amongst the genes of the K-means clusters

Cluster [genes]	InterPro ID	InterPro Description	Freq. in total [6142]	Occur. In total [6142]	Freq. in cluster	Occur. in cluster	P-value
M1 [96]	IPR006629	LPS-induced tumor necrosis factor	4.9E-04	3	0.021	2	7.1E-04*
	IPR002068	Heat shock protein Hsp20	6.5E-04	4	0.021	2	0.001*
	IPR001436	Alpha crystallin/Heat shock protein	6.5E-04	4	0.021	2	0.001*
	IPR001304	C-type lectin	0.002	15	0.031	3	0.001*
M2 [333]	IPR013154	Alcohol dehydrogenase GroES-like	0.001	8	0.012	4	4.8E-04*
	IPR002115	Protein-tyrosine phosphatase	3.3E-04	2	0.006	2	0.003*
	IPR001148	Carbonic anhydrase, eukaryotic	0.001	7	0.009	3	0.004*
	IPR002048	Calcium-binding EF-hand	0.010	64	0.027	9	0.005*
M3 [91]	IPR000435	Tektin	4.9E-04	3	0.033	3	3.1E-06 *
	IPR006602	Region of unknown function DM10	3.3E-04	2	0.023	2	2.2E-04 *
	IPR000008	C2 calcium-dependent targeting	0.004	26	0.044	4	4.9E-04
	IPR001565	Synaptotagmin	8.1E-04	5	0.022	2	0.002
M4 [92]	IPR008011	Complex 1 LYR protein	4.9E-04	3	0.033	3	3.3E-06 *
	IPR000348	emp24/gp25L/p24	8.1E-04	5	0.022	2	0.002*
	IPR009038	GOLD	9.8E-04	6	0.022	2	0.003*
F1 [156]	IPR007109	Brix domain	6.5E-04	4	0.019	3	6.3E-05 *
	IPR006984	Unknown function DUF652	3.3E-04	2	0.013	2	6.4E-04*
	IPR007125	Histone core	0.001	8	0.019	3	7.9E-04*
	IPR001680	WD40 repeat	0.019	119	0.051	8	0.007
F2 [726]	IPR014021	Helicase, 1 and 2, ATP-binding	0.009	55	0.032	23	1.3E-08 *
	IPR014001	DEAD-like helicase, N-terminal	0.011	68	0.034	25	6.3E-08 *
	IPR001844	Chaperonin Cpn60	9.8E-04	6	0.008	6	2.7E-06 *
	IPR000795	Protein synthesis factor	0.002	11	0.011	8	4.2E-06 *
F3 [88]	IPR000585	Hemopexin	9.8E-04	6	0.023	2	0.003*
	IPR013525	ABC-2 type transporter	0.001	7	0.02	2	0.004*
	IPR001506	Peptidase M12A, astacin	0.001	8	0.02	2	0.005*
	IPR006195	Aminoacyl-tRNA synthetase II	0.002	11	0.02	2	0.010*
F4 [151]	IPR003960	ATPase, AAA-type, conserved site	0.002	12	0.026	4	1.4E-04 *
	IPR000991	Glutamine amidotransferase class-I	3.3E-04	2	0.013	2	6.0E-04*
	IPR002318	Alanyl-tRNA synthetase, class IIc	3.3E-04	2	0.013	2	60E-04*
	IPR013315	Spectrin alpha chain, SH3 domain	0.001	9	0.020	3	0.001*
E1 [14]	IPR001007	von Willebrand factor, type C	0.001	8	0.143	2	1.3E-04 *
E2 [9]	IPR002194	AAA+ ATPase, core	0.017	104	0.222	2	0.009*
E3 [34]	IPR001599	Alpha-2-macroglobulin	0.001	9	0.176	6	1.5E-12 *
	IPR008068	Cytochrome P450, E-class, group I	4.9E-04	3	0.059	2	8.9E-05 *
	IPR000875	Cecropin	6.5E-04	4	0.059	2	1.8E-04 *
	IPR013053	Hormone binding	0.001	8	0.059	2	8.1E-04 *

* Statistically significant overrepresentation of domain name according to Bonferroni corrected hypergeometric distribution.
