# Supplementary material for: Transcription Regulation of Sex-Biased Genes during Ontogeny in the Malaria Vector Anopheles gambiae
Source: PLoS One. 2011 Jun 30;6(6):e21572. doi: 10.1371/journal.pone.0021572 (PMC3128074; doi:10.1371/journal.pone.0021572)
Supplement: Table S9 — Observed and expected frequency of unique sex-biased genes in A. gambiae. (PDF) [file pone.0021572.s017.pdf]

Table S9

Observed and expected frequency of unique sex-biased genes in *A. gambiae*

		Total	Obs. unique genes	Exp. unique genes	% (>/<)	P- value
Larvae						
An:Dm (6134 genes / 1088 unique)	Male biased	142	21	22.3	-6.0%	0.09
	Female biased	31	11	4.9	125.5%	4.0E-3*
An:Ae (6134 genes / 686 unique)	Male biased	142	26	14.0	86.3%	6.7E-4*
	Female biased	31	10	3.0	228.3%	4.1E-4*
An:Ae:Dm (6136 genes / 385 unique)	Male biased	142	9	7.3	24%	0.11
	Female biased	31	4	1.6	152.4%	0.05
Pupae						
An:Dm (4494 genes / 666 unique)	Male biased	65	13	9.6	35.0%	0.06
	Female biased	45	15	6.7	124.9%	9.7E-4*
An:Ae (4475 genes / 432 unique)	Male biased	65	12	6.2	92.1%	0.01
	Female biased	45	9	4.3	108.1%	0.01
An:Ae:Dm (4475 genes / 220 unique)	Male biased	65	5	3.2	57.1%	0.11
	Female biased	45	7	2.2	217.8%	4.4E-3*
Adult						
An:Dm (5033 genes / 818 unique)	Male biased	629	179	102.2	75.1%	~0*
	Female biased	1123	119	182.5	-34.8%	4.3E-10*
An:Ae (5033 genes / 511 unique)	Male biased	629	91	63.9	42.5%	6.2E-5*
	Female biased	1123	93	114.0	-18.4%	2.6E-3
An:Ae:Dm (5028 genes / 283 unique)	Male biased	629	69	35.4	95.1%	~0*
	Female biased	1123	42	63.1	-33.5%	3.5E-4*

* Statistically significant overrepresentation according to Bonferroni corrected hypergeometric distribution.
