# Supplementary material for: Transcription Regulation of Sex-Biased Genes during Ontogeny in the Malaria Vector Anopheles gambiae
Source: PLoS One. 2011 Jun 30;6(6):e21572. doi: 10.1371/journal.pone.0021572 (PMC3128074; doi:10.1371/journal.pone.0021572)
Supplement: Table S10 — Observed and expected frequency of unique sex biased genes within individual K-means clusters. (PDF) [file pone.0021572.s018.pdf]

Table S10

Observed and expected frequency of unique sex biased genes within individual k-means clusters.

	**Total**	**Obs. unique genes**	**Exp. unique genes**	**% (>/<)**	**P- value**
An:Dm (6142 genes /1091 unique)					
M1	96	33	17.1	93.5%	3.9E-05*
M2	333	104	59.2	75.8%	3.0E-10*
M3	91	15	16.2	7.2%	0.11
M4	92	14	16.3	14.3%	0.09
F1	156	25	27.7	9.8%	0.07
F2	726	73	129.0	43.4%	3.1E-10*
F3	88	6	15.6	61.6%	1.8E-03*
F4	151	14	26.8	47.8%	1.3E-03*
E1	14	1	2.5	59.8%	0.20
E2	9	2	1.6	25.1%	0.29
E3	34	11	6.0	82.1%	0.02
An:Ae (6142 genes /688 unique)					
M1	96	16	10.8	48.8%	0.03
M2	333	55	37.3	47.4%	7.0E-04*
M3	91	11	10.2	7.9%	0.12
M4	92	7	10.3	32.1%	0.08
F1	156	11	17.5	37.1%	0.03
F2	726	70	81.3	13.9%	0.02
F3	88	9	9.9	8.7%	0.13
F4	151	5	16.9	70.4%	2.8E-04*
E1	14	3	1.6	91.3%	0.14
E2	9	1	1.0	0.8%	0.39
E3	34	4	3.8	5.0%	0.21
An:Ae:Dm (6142 genes /386 unique)					
M1	96	12	6.0	98.9%	0.01
M2	333	48	20.9	129.4%	1.9E-08*
M3	91	4	5.7	30.1%	0.15
M4	92	5	5.8	13.5%	0.17
F1	156	8	9.8	18.4%	0.12
F2	726	30	45.6	34.2%	2.1E-03
F3	88	3	5.5	45.8%	0.11
F4	151	2	9.5	78.9%	2.6E-03*
E1	14	1	0.9	13.7%	0.38
E2	9	1	0.6	76.8%	0.34
E3	34	2	2.1	6.4%	0.28

* Statistically significant overrepresentation according to Bonferroni corrected hypergeometric distribution.
