# Supplementary material for: Transcription Regulation of Sex-Biased Genes during Ontogeny in the Malaria Vector Anopheles gambiae
Source: PLoS One. 2011 Jun 30;6(6):e21572. doi: 10.1371/journal.pone.0021572 (PMC3128074; doi:10.1371/journal.pone.0021572)
Supplement: Table S11 — List of genes with a conserved male-biased transcription profile in adult A. gambiae and D. melanogaster. (PDF) [file pone.0021572.s019.pdf]

Table S11
List of genes with a conserved male-biased transcription profile in adult *A. gambiae* and *D. melanogaster*

***A. gambiae* Gene ID**	***Drosophila* orthologue Gene ID**	***Drosophila* Testes**	***Drosophila* Carcass**
AGAP001620	CG10252	+	-
AGAP001735*	CG7264	+	-
AGAP002365	CG10841	+	-
AGAP003227**	CG8362	+	-
AGAP003238	CG2082	+	+
AGAP004030	CG6971	+	-
AGAP004068	CG10999	+	+
AGAP004621*	CG17083	+	-
AGAP004622	CG4434	+	-
AGAP008338	CG5280	+	-
AGAP008689	CG9313	+	-
AGAP009072	CG8680	+	+
AGAP009084*	CG5048	+	-
AGAP010031*	CG4767	+	-
AGAP010199*	CG5458	+	-
AGAP010341	CG14305	+	-
AGAP010326	CG5398	+	-
AGAP010786	CG18472	+	-

* Transcribed exclusively in the *A. gambiae* testis

** Transcribed exclusively in both the testis and the ovaries
