# Supplementary material for: Transcription Regulation of Sex-Biased Genes during Ontogeny in the Malaria Vector Anopheles gambiae
Source: PLoS One. 2011 Jun 30;6(6):e21572. doi: 10.1371/journal.pone.0021572 (PMC3128074; doi:10.1371/journal.pone.0021572)
Supplement: Methods S1 — (DOC) [file pone.0021572.s021.doc]

Methods S1

For the 454 sequencing analysis, cDNA was produced from sexed larvae using the supersmart cDNA synthesis kit (Stratagene #635000) which allows the production of large amounts of cDNA from limiting quantities of total RNA through linear PCR amplification of full length cDNAs. Double stranded cDNA samples were fragmented by nebulization and prepared for pyrosequencing, performed using Titanium Genome Sequencer platform (Roche/454 Life Sciences, Branford, CT). Average read length was approximately 400bp and SCARF (Barker et al. 2009), an open source software based on NCBI’s megablast, designed specifically for mapping 454 reads against a reference dataset, was used to map our reads against the reference transcript dataset from *A. gambiae* (Vectorbase ,AgamP3.3). Over 200,000 mappable reads were obtained for each condition. To compare transcript levels of different genes, reads were quantified in reads per kilobase of exon model, similar to the method proposed in Mortazavi et al (2008).

Barker M.S. et al Bioinformatics 25, 535 (Feb 15, 2009).

Mortazavi et al Nat Methods 2008;5:621-628.
